# Comparison of genome-wide array genomic hybridization platforms for the detection of copy number variants in idiopathic mental retardation

**DOI:** 10.1186/1755-8794-4-25

**Published:** 2011-03-25

**Authors:** Tracy Tucker, Alexandre Montpetit, David Chai, Susanna Chan, Sébastien Chénier, Bradley P Coe, Allen Delaney, Patrice Eydoux, Wan L Lam, Sylvie Langlois, Emmanuelle Lemyre, Marco Marra, Hong Qian, Guy A Rouleau, David Vincent, Jacques L Michaud, Jan M Friedman

**Affiliations:** 1 Department of Medical Genetics, University of British Columbia, Vancouver, British Columbia, Canada; 2 McGill University and Genome Quebec Innovation Centre, Montréal, Quebec, Canada; 3 Children's & Women's Hospital, Vancouver, British Columbia, Canada; 4 Genome Sciences Centre, BC Cancer Agency, Vancouver, British Columbia, Canada; 5 CHU Sainte-Justine Research Center, Montréal, Quebec, Canada; 6 British Columbia Cancer Research Centre, Vancouver, British Columbia, Canada; 7 Child & Family Research Institute, Vancouver, British Columbia, Canada; 8 Center of Excellence in Neuromics of Université de Montréal, Montréal, Quebec, Canada; 9 CHUM Research Center, Montréal, Quebec, Canada

## Abstract

**Background:**

Clinical laboratories are adopting array genomic hybridization as a standard clinical test. A number of whole genome array genomic hybridization platforms are available, but little is known about their comparative performance in a clinical context.

**Methods:**

We studied 30 children with idiopathic MR and both unaffected parents of each child using Affymetrix 500 K GeneChip SNP arrays, Agilent Human Genome 244 K oligonucleotide arrays and NimbleGen 385 K Whole-Genome oligonucleotide arrays. We also determined whether CNVs called on these platforms were detected by Illumina Hap550 beadchips or SMRT 32 K BAC whole genome tiling arrays and tested 15 of the 30 trios on Affymetrix 6.0 SNP arrays.

**Results:**

The Affymetrix 500 K, Agilent and NimbleGen platforms identified 3061 autosomal and 117 X chromosomal CNVs in the 30 trios. 147 of these CNVs appeared to be *de novo*, but only 34 (22%) were found on more than one platform. Performing genotype-phenotype correlations, we identified 7 most likely pathogenic and 2 possibly pathogenic CNVs for MR. All 9 of these putatively pathogenic CNVs were detected by the Affymetrix 500 K, Agilent, NimbleGen and the Illumina arrays, and 5 were found by the SMRT BAC array. Both putatively pathogenic CNVs identified in the 15 trios tested with the Affymetrix 6.0 were identified by this platform.

**Conclusions:**

Our findings demonstrate that different results are obtained with different platforms and illustrate the trade-off that exists between sensitivity and specificity. The large number of apparently false positive CNV calls on each of the platforms supports the need for validating clinically important findings with a different technology.

## Background

Chromosomal abnormalities, the most frequently diagnosed cause of mental retardation (MR)[1], are routinely identified by cytogenetic analysis. Studies using array genomic hybridization (AGH) have found apparently-pathogenic gains or losses of genetic material in at least 10% of children with MR and normal conventional cytogenetic analysis[2-4]. These apparently pathogenic deletions and duplications range in size from < 100 Kb to 15 Mb. Such submicroscopic chromosomal gains or losses are collectively called pathogenic copy number variants (CNVs).

However, most CNVs, despite producing genomic imbalance of many thousands of DNA base pairs, do not cause MR. In fact, CNVs are the greatest source of genetic variation in normal people; the mean number of apparently benign CNVs observed ranges from 10's-1000's per person, depending on the technology used[5-9]. Distinguishing benign CNVs from those that cause MR and other birth defects is the most serious challenge to the routine clinical use of AGH, especially for prenatal diagnosis[4,10-13].

A consensus has developed that AGH should be offered routinely in the evaluation of children with MR and other birth defects[2,3,11]. However, there is no agreement regarding the choice of AGH platform, resolution, or reference sample that is most appropriate for clinical use[3,14-17]. AGH for clinical diagnosis has often employed targeted arrays with probes in genomic regions known to be associated with microdeletion and microduplication syndromes, and more recent versions of many targeted arrays have added additional probes (i.e., a 'backbone') to provide some degree of genome-wide coverage. As the density of probes in these backbones increases, genome-wide and targeted platforms are converging, with both providing a survey of the whole genome at relatively high resolution.

Few studies have compared AGH whole genome technologies[18-20], and most are retrospective and limited to pathogenic CNVs. We performed AGH studies on 30 MR trios (children with idiopathic MR and both of their unaffected parents) using three different high-resolution genome-wide oligonucleotide platforms -- Affymetrix 500 K, Agilent 244 K and NimbleGen 385 K -- to assess their utility for the identification of pathogenic CNVs in children with MR. We determined whether the CNVs called on these platforms were also detected by the Illumina Hap550 Beadchip or the SMRT 32 K BAC whole genome tiling array and tested samples from 15 of the MR trios on Affymetrix 6.0 arrays. This large comparison of multiple AGH platforms provides unique insights into both the power and the limitations of current technology for detecting pathogenic genomic imbalance in children with MR.

## Methods

### Patients

Patients with MR and at least one of the following additional characteristics were selected for study: 1) growth retardation of pre- and/or post-natal onset; 2) microcephaly or macrocephaly; 3) one or more major malformations, and 4) more than two facial dysmorphic features. Characterization of this cohort with the checklist developed by de Vries et al.[21] showed an average score of 4.4 (range: 2-9). The cause of the MR in each child was unknown despite full evaluation by a clinical geneticist, a karyotype at ≥500 band resolution and subtelomeric FISH studies. This study was approved by the University of British Columbia Clinical Research Ethics Board and Hopital Sainte-Justine Research Ethics Board, and informed consent was obtained from each family.

### Array Genomic Hybridization

Genomic DNA was extracted from blood samples using the Puregene DNA kit (Gentra System). DNA quality for each trio was assessed by electrophoresis in a 1% agarose gel, and DNA concentration was measured with a NanoDrop™ Spectrophotometer.

AGH was performed on 30 children with idiopathic MR and on both normal parents of each child on Affymetrix 500 K GeneChips, Agilent 244 K Oligonucleotide Arrays, and NimbleGen 385 K Oligonucleotide Arrays. In addition, DNA from the 30 children was run on Illumina Hap550 Beadchips using a set of about 100 HapMap samples as reference. DNAs from the 30 children were also run on Sub-Megabase Resolution Tiling-set (SMRT) human genomic BAC arrays using one of the parents - the one of the same sex - as reference. In addition, DNA samples from 15 trios were run on the Affymetrix Genome-Wide Human SNP Array 6.0.

All samples were handled according to the platform manufacturer's recommendations, and CNV detection was performed using the manufacturer's recommended software with default settings (See Additional File for detailed protocol and software settings).

### Identification of Autosomal *de novo *Changes

A CNV was considered to be *de novo *on Affymetrix 500 K, Agilent 244 K, NimbleGen 385 K, or Affymetrix 6.0 AGH if a set of probes identified by the platform algorithm was called as a deletion in the child relative to both parents or as a duplication in the child relative to both parents on the same platform.

### Identification of X Chromosomal CNVs

In addition to looking for *de novo *CNVs as described for the autosomes above, we performed manual assessments for CNVs when there was a sex-mismatch between the child and parent because the NimbleGen and Affymetrix platforms are unable to account for sex mismatches between the test and reference DNAs.

### Identification of Pathogenic Changes

We used *de novo *occurrence as a major criterion of pathogenicity for autosomal CNVs; however, most *de novo *changes found in these studies are unlikely to be pathogenic for MR in the children studied. In addition, all X chromosomal CNVs except those in the pseudoautosomal or XY homology regions were assessed for pathogenicity. We used previously published criteria[4,12,13,22,23] to determine which CNVs are likely to be pathogenic. We performed genotype-phenotype correlations only on CNVs that were greater than 50 Kb in length and that met the criteria of pathogenicity cited above.

### CNV Confirmation

CNVs were validated by FISH, MLPA, qPCR or PCR (for X chromosome deletions identified in males). See Additional File 1 for detailed validation protocols.

### Statistical Analysis

A chi square analysis was performed to assess differences between platforms in the proportion of singleton CNV calls and the number of *de novo *CNVs. Differences in sizes and types of CNVs called between platforms were assessed by Mann-Whitney U tests. A p-value of 0.05 was considered significant in all analyses.

## Results

We compared the ability of the Affymetrix 500 K, Agilent 244 K and NimbleGen 385 K platforms to detect *de novo *CNVs in 30 patients with idiopathic MR using the normal parents of each child as reference.

**Figure 1:**
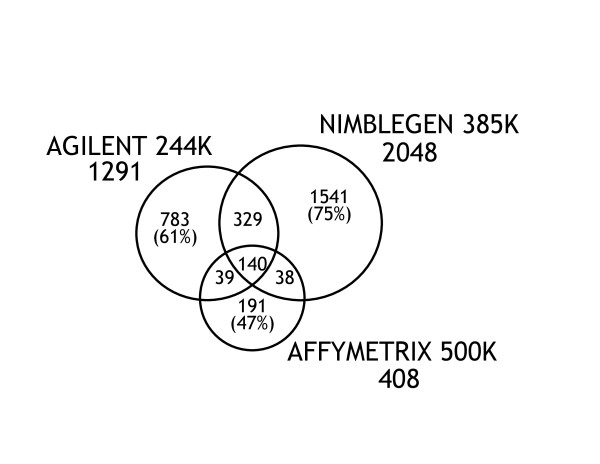
**Venn diagrams of CNV calls made by the 3 main AGH platforms**. The numbers under each platform name indicate the total number of CNV calls by that platform. The numbers in the intersecting regions indicate CNV calls made by multiple platforms. The numbers outside the intersecting regions are the number of CNVs that were unique to that platform.

Overall, 1,492 autosomal deletions and 1,569 autosomal duplications were called in the 30 children with MR by one or more of the three main platforms (Affymetrix 500 K, Agilent 244 K, and NimbleGen 385 K; Additional File 2 and Additional File 1). Over 80% of the autosomal CNV calls were made by only one of the three major platforms. The proportion of singleton CNV calls made was significantly different among the 3 platforms (p = < 0.001). The NimbleGen platform identified about 40% more autosomal calls than the Agilent platform, while the higher density Affymetrix 500 K platform identified fewer than one third as many CNVs as the Agilent platform (Figure 1). However, 60% of the autosomal CNVs identified only on the Agilent or NimbleGen platform are in genomic regions that had fewer than 5 probes on the Affymetrix 500 K array, so recognition of such CNVs would not be expected.

### Detection of autosomal *de novo *CNVs

There were 146 autosomal *de novo *CNVs identified on one or more of the three main platforms in the 30 MR probands, an average of 4.9 *de novo *CNVs per patient (Additional File 3). Two patients had only one *de novo *CNV identified, 3 had 2 *de novo *CNVs, 5 had 3 *de novo *CNVs and 20 patients had 4 or more *de novo *CNVs.

**Figure 2:**
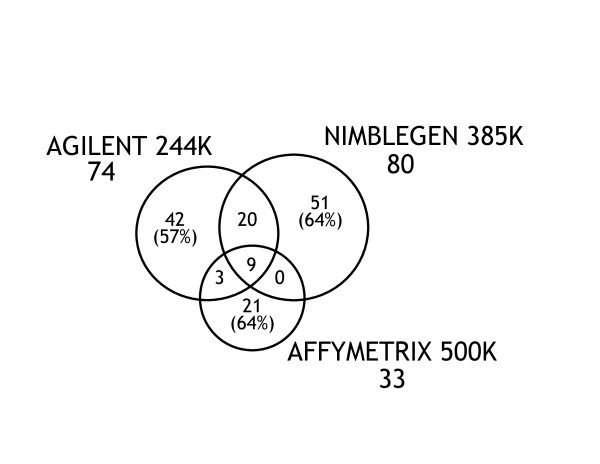
**Venn diagrams of autosomal *de novo *CNV calls made by the 3 main AGH platforms**. The numbers under each platform name indicate the total number of *de novo *CNV calls by that platform. The numbers in the intersecting regions present CNV calls made by multiple platforms. The numbers outside the intersecting regions are the number of CNVs that were unique to that platform.

114 (78%) of the autosomal *de novo *calls were identified on only one platform, 23 on 2 platforms and 9 on all 3 platforms (Figure 2). Significantly fewer *de novo *calls were made with the Affymetrix 500 K system than with Agilent (p = < 0.001) or NimbleGen platforms (p = < 0.001).

A larger number of autosomal *de novo *deletions (96) than duplications (50) were called. The median size of the *de novo *deletions (113 Kb) was similar to that of the *de novo *duplications (98 Kb) (p = 0.348). The median size of the *de novo *CNVs detected by the NimbleGen platform (183 Kb) was significantly larger than those identified by the Agilent platform (82 Kb; p = 0.003) but not significantly larger than those identified by the Affymetrix 500 K platform (145 Kb; p = 0.083; Additional File 1).

Many autosomal *de novo *CNVs were observed in multiple individuals in this small series. These recurrent calls accounted for 45 (31%) of the 146 autosomal *de novo *CNVs called in the 30 trios, and all occurred in regions that contain polymorphic CNVs previously recognized by oligonucleotide arrays or higher resolution techniques (Database of Genomic Variants (DGV), http://projects.tcag.ca/variation/) (Additional File 3).

### Detection of autosomal *de novo *CNVs with the Affymetrix 6.0 Platform

To explore whether the higher density Affymetrix 6.0 array improved CNV detection in comparison to the three main platforms, we arbitrarily selected 15 of the 30 MR trios for analysis using the Affymetrix 6.0 platform (see Additional File 1 for details of analysis).

There was a total of 915 autosomal CNVs identified with the Affymetrix 6.0 platform in the 15 probands (an average of 61 CNVs per person) (Additional File 4), compared to 281 autosomal CNVs (an average of 18.7 per person) in these same 15 individuals on the Affymetrix 500 K platform. 682 CNVs were called in these 15 probands on the Agilent platform and 973 CNVs were called on the NimbleGen platform.

41 of the 915 autosomal CNVs identified by Affymetrix 6.0 AGH occurred *de novo*, and 29 of these 41 (71%) CNVs were not found by any of the three main platforms. The Affymetrix 6.0 platform identified 9 of 12 CNVs that were called as *de novo *on 2 or 3 of the main platforms in these patients (Additional File 3).

### Detection of autosomal *de novo *CNVs with the Illumina and SMRT Platforms

In more limited comparisons, we sought to determine whether autosomal *de novo *CNVs identified by the three main platforms studied are likely to be identified by the Illumina Hap550 Beadarray or the SMRT 32 K BAC tiling array.

The Illumina platform identified 14 of the 146 autosomal *de novo *CNVs that had been called on one or more of the 3 main platforms and 8 of the 9 *de novo *CNVs that had been called on all 3 main platforms (Additional File 3). All Illumina CNV calls are listed in Additional File 5.

The SMRT platform identified 9 of the 146 autosomal *de novo *CNVs called on one or more of the 3 main platforms, including 4 of the 9 *de novo *CNVs called on all 3 of the main platforms (size range: 333 Kb-9.8 Mb). All CNVs called by SMRT AGH are listed in Additional File 6.

### Detection of X chromosomal CNVs

The X chromosome was analysed separately because half of our hybridizations are sex-mismatched and the Affymetrix and NimbleGen CNV detection software are unable to correct for this. Therefore, identifying CNVs in sex-mismatched hybridizations on these platforms required manual assessment, a process that is inherently more subjective than the automated assessment used for the other platforms.

**Figure 3:**
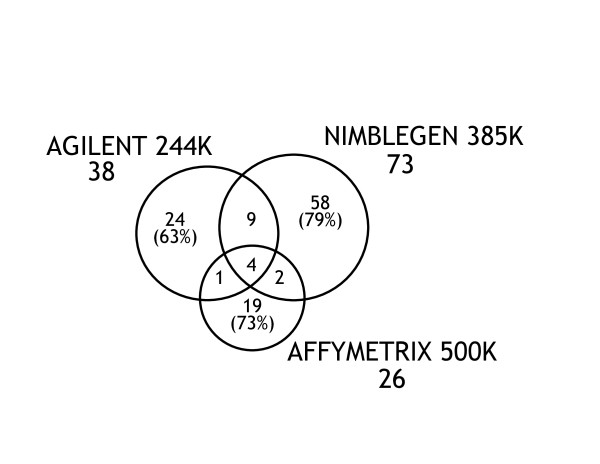
**Venn diagram of X chromosome CNV calls made by the 3 main AGH platforms**. The numbers under each platform name indicate the total number of CNV calls by that platform. The numbers in the intersecting regions present CNV calls made by multiple platforms. The numbers outside the intersecting regions are the number of CNVs that were unique to that platform.

There were 117 X-chromosomal CNVs identified on one or more of the 3 main platforms in these 30 MR trios (Additional File 7). 23 of these 117 CNVs were identified in 9 females. 101 (86%) of the CNVs were identified by only 1 platform (Figure 3).

Fewer X-chromosomal CNV calls were made on the Agilent (38) and Affymetrix 500 K (26) platforms than on the NimbleGen platform (73). A larger number of deletions (78) than duplications (39) were identified on the X chromosome. The median size of the deletions (131 Kb) was similar to that of the duplications (97 Kb, p = 0.175). The median size of the X-chromosomal CNVs detected by the Agilent platform (32 Kb) was significantly smaller than that of the CNVs identified by the Affymetrix 500 K (209 Kb, p < 0.001) or NimbleGen (168 Kb, p < 0.001) platforms.

Four of the 117 X-chromosomal CNVs called on the 3 main platforms were also identified by the Illumina platform, 6 by the Affymetrix 6.0 platform and 1 by the SMRT BAC platform (Additional File 7).

### Autosomal *de novo *CNVs with Potential Clinical Significance

In a clinical laboratory, it is not usually possible to use multiple platforms to determine which CNV calls are real, and only a subset of the CNVs called with any technology is likely to be pathogenic. We used previously published criteria to determine which CNVs identified by one or more of the Agilent, NimbleGen, Affymetrix 500 K and Affymetrix 6.0 platforms are likely to be pathogenic (Additional File 4).

Only autosomal CNVs that occurred *de novo *were assessed for pathogenicity. Although inherited CNVs may cause MR[24-27], they are much less likely to do so than *de novo *CNVs, and there was no clinical reason to suspect a pathogenic inherited CNV in any of these children.

Smaller CNVs are much more likely than large CNVs to be false positives and to be benign rather than pathogenic[23]. Therefore, we restricted the analysis for likely pathogenicity to *de novo *CNVs that were 50 Kb or larger. This eliminated from consideration 51 CNVs (36 deletions and 15 amplifications) that were < 50 Kb.

We also eliminated from consideration 21 *de novo *CNVs (13 deletions and 8 duplications) that did not contain validated open reading frames because such CNVs cannot be interpreted as pathogenic unless they involve a non-coding region known to be associated with MR. We eliminated a further 30 *de novo *CNVs (20 deletions and 10 duplications) that occurred in regions that contain only genetically unstable highly repetitive genes such as olfactory receptor genes or immunoglobin genes. In addition, given the small sample size, any *de novo *CNV that was identified in 4 or more probands with differing phenotypes and that was not known to be pathogenic for MR was deemed unlikely to be pathogenic, and we eliminated an additional 19 CNVs (8 deletions and 11 duplications) for this reason.

We subjected 21 of the remaining 25 *de novo *CNVs identified in 14 individuals to FISH or MLPA validation. Eight of these *de novo *CNVs were called by all 3 of the main platforms, and all 8 were confirmed by FISH or MLPA. In contrast, the 11 *de novo *CNVs identified on just one of the 3 main platforms and two *de novo *CNVs identified by two of the 3 main platforms could not be validated by FISH or MLPA.

### X Chromosomal CNVs with Potential Clinical Significance

We considered 31 of the 117 X-chromosomal CNVs that were less than 50 Kb and 21 that did not contain open reading frames unlikely to be pathogenic and eliminated them from further assessment. 32 other X-chromosomal CNVs were recurrent within this study population and were also eliminated from further analysis. One 7 Mb *de novo *amplification in a male was validated by FISH (Patient 8960) and was identified by all 3 of the main platforms. 17 other CNVs were tested with PCR or qPCR and could not be confirmed (Additional File 1). These 17 CNVs were eliminated from further consideration as being pathogenic.

### Genotype-Phenotype Correlations

Of the 28 remaining *de novo *CNVs, 12 were autosomal and 16, X-linked. We considered 4 autosomal *de novo *CNVs (Table 1) and 11 X chromosomal CNVs (Table 2) to be unlikely to cause MR because they contained no RefSeq genes that appeared to be reasonable candidates for pathogenicity. Two X-chromosomal CNVs result in a MR phenotype in males but not in females[28,29], and both were eliminated from further consideration because they occurred in a female (Patient 7093). One X chromosomal deletion in a male (Patient 3094) was eliminated because it results in MR when deleted in females but not in males[30]. Another maternally inherited duplication was eliminated in a female (Patient 1815) because only deletions have been reported to result in MR in females[31].

**Table 1:** Summary of autosomal *de novo *CNVs identified on the three main AGH platforms selected for genotype-phenotype analysis.

Trio ID	Chr	CNV Type	Start*	Size*	Platforms Identified CNV	# of RefSeq Genes	Validation	Comment on Gene Function
1815	3	DEL	196 904 149	54 518	Agilent	1	NT	Mucin 20 - Expression pattern not consistent with causing MR[40]

4821	5	DEL	68 950 015	1 329 642	NimbleGen	7	NT	Mutations in SMN1 associated with spinal muscle atrophy[41]

8960	5	DUP	180 309 941	55 922	NimbleGen	2	MLPA Pos	Expression pattern not consistent with causing MR[42]

1815	6	DEL	111 807 663	9 889 630	All 3 platforms	57	FISH Pos	Likely pathogenic based on size

7531	9	DEL	139 496 489	333 935	All 3 platforms	7	FISH Pos	CNVs in region previously reported as pathogenic[32]

1815	12	DEL	11 371 263	83 667	NimbleGen	1	MLPA Pos	Expression pattern not consistent with causing MR[43]

1056	13	DEL	107 190 506	2 206 948	All 3 platforms	5	FISH Pos	Encompassed within *de novo *CNV in DECIPHER patient with MR

4821	16	DEL	3 862 993	78 891	All 3 platforms	1	MLPA Pos	CNVs in region previously reported as pathogenic[35]

3921	17	DEL	41 062 469	657 364	All 3 platforms	8	FISH Pos	CNVs in region previously reported as pathogenic[33]

9609	21	DEL	33 902 218	152 885	All 3 platforms	2	MLPA Pos	Important in spinal development[37]

9609	22	DEL	19 062 809	728 798	All 3 platforms	19	FISH Pos	CNVs in region previously reported as pathogenic[34,44]

8327	22	DUP	19 412 033	378 797	All 3 platforms	13	MLPA Pos	Mutation has been reported in family with normal phenotype[25]

DEL = Deletion; DUP = Amplification, NT - Not tested; Pos = Positive; Neg = Negative; N/T = Not tested

*Start/end coordinates determined from largest region of overlap between any two platforms; size is the difference between these two coordinates (Build 36).

**Table 2:** Summary of X chromosome CNVs identified on the three main AGH platforms selected for genotype-phenotype analysis.

Trio ID	CNV Type	Start*	Size*	Platforms Identified CNV	Genes Involved	Validation	Comment on Gene Function
6428	DEL	148 264 112	156 992	Affy	IDS	NT	Expression pattern not consistent with causing MR

2894	DEL	101 266 713	250 045	NimbleGen	5 RefSeq genes	NT	Expression pattern not consistent with causing MR[45]

3519	DEL	9 454 329	197 920	Affy	TBL1X	NT	Expression pattern not consistent with causing MR[46]

8960	DUP	67 416 262	7 057 217	All 3 platforms	57 RefSeq genes	FISH Pos	FISH Pos

9313	DEL	9 484 049	165 559	Affy	TBL1X	NT	Expression pattern not consistent with causing MR

3921	DUP	74 811 330	208 698	NimbleGen	TTC3L	NT	Expression pattern not consistent with causing MR[47]

1511	DEL	6 856 649	201 556	Affy	HDHD1A	NT	Expression pattern not consistent with causing MR[48]

2714	DEL	76 534 899	67 182	NimbleGen	FGF16	NT	Expression pattern not consistent with causing MR[49]

5993	DEL	6 932 549	130 699	NimbleGen	HDHD1A	NT	Expression pattern not consistent with causing MR[48]

4821	DEL	6 625 133	419 230	NimbleGen	HDHD1A	NT	Expression pattern not consistent with causing MR[48]

3921	DUP	74 811330	208 698	NimbleGen	MAGEE2	NT	Expression pattern not consistent with causing MR[47]

8960	DEL	29 967 317	203 809	Affy	MEGB2E	NT	Expression pattern not consistent with causing MR[50]

1815	DUP	67 767 923	2 019 581	NimbleGen	15 RefSeq genes including DLG3	NT	Amplification not reported to cause MR[31]

7093	DUP	73 429 587	263 642	NimbleGen	3 RefSeq genes including SLC16A2	NT	Females not affected[28]

3094	DEL	99 293 227	205 443	Affy	PCDH19	NT	Males not affected[30]

7093	DEL	6 687 308	906 505	Agilent & NimbleGen	STS	NT	Females not affected[29]

DEL = Deletion; DUP = Amplification, NT - Not tested; Pos = Positive; Neg = Negative; N/T = Not tested

*Start/end coordinates determined from largest region of overlap between any two platforms; size is the difference between these two coordinates (Build 36).

Of the remaining 9 *de novo *CNVs, 7 are likely to be pathogenic. Table 3 summarizes the phenotypes of these patients. The CNVs that are likely to be pathogenic include deletions within 9q34.3 (Patient 7531), 17q21.31 (Patient 3921) and 22q11.2 (Patient 9609) that have been previously reported to be pathogenic in other patients with similar phenotypes[32-34]. A 9.8 MB deletion (Patient 1815) and 7 Mb duplication (Patient 8960) are likely to be pathogenic based on their size and the number of genes affected. Patient 4821 has a 78 Kb deletion within the first exon and upstream sequence of the CREB binding protein (*CREBBP*), haploinsufficiency of which causes the Rubinstein-Taybi syndrome[35]. The phenotype of Patient 4821 is consistent with this diagnosis. The seventh patient has a previously undescribed 2.2 Mb deletion of chromosome 13q11 (Patient 1056) that encompasses 5 genes, including myosin *16 (MYO16)*, which codes for a protein that interacts with known synaptic proteins that are important for cognition[36]. The deletion in this patient falls within a 9.9 Mb *de novo *deletion in another patient with MR who is listed in DECIPHER (DECIPHER patient ID 4668).

**Table 3:** Summary of phenotypes in patients with validated pathogenic or possibly pathogenic CNVs

ID	Chr	CNV	Start*	Size*	Pathogenicity	Phenotype
1815	6	DEL	111 807 663	9 889 630	Likely	MR, microcephaly, epicanthic folds, small ears, hypoplastic lobes, micrognathia, brachycephaly, hypotonia

7531	9	DEL	139 496 489	333 935	Likely	Moderate global developmental delay, microcephaly, flat face, upslanting palpebral fissures, hypertelorism, synophrysm, anteverted nares, hypoplasia of the amygdalo-hippocampic complex

1056	13	DEL	107 190 506	2 206 948	Likely	Moderate MR, upslanting palpebral fissures, retrognathia

4821	16	DEL	3 862 993	78 891	Likely	Moderate MR, microcephaly, short stature, bilateral glaucoma, bilateral colobomas of the optic nerves, neuro-sensorial deafness, large ASD, epicanthic folds, low nasal septum, preauricular pits, low set ears, broad distal phalanges of all fingers and toes, cryptorchidy, hypotonia

3921	17	DEL	41 062 469	657 364	Likely	Mild MR, sagittal craniosynostosis, malar hypoplasia, mild retrognathia, short and upslanting palpebral fissures, low set ears, high arched palate, broad proximal phalangeal joints of the hands, unilateral cryptorchidism

9609	22	DEL	19 062 809	728 798	Likely	Moderate MR, microcephaly, short stature, down-slanting palpebral fissures, low-set ears, wide nasal base, retrognathia, metopic craniosynostosis, cleft palate, partial agenesis of the corpus callosum, tetralogy of Fallot

8960	X	DUP	67 416 262	7 057 217	Likely	Moderate MR, brachycephaly, bilateral epicanthic folds, posteriorly rotated ears with hypoplastic helix and hypotnic

9609	21	DEL	33 902 218	152 885	Possible	See above pathogenic mutation

8327	22	DUP	19 412 033	378 797	Possible	Mild MR, small stature, Pierre Robin sequence with cleft palate

DEL = Deletion; DUP = Amplification

*Start/end coordinates determined from largest region of overlap between any two platforms; size is the difference between these two coordinates (Build 36).

These 7 *de novo *CNVs that are likely to be pathogenic were all identified on all three of the main AGH platforms studied - Agilent, NimbleGen and Affymetrix 500 K - as well as on the Illumina platform. Five of these 7 cases were also identified by SMRT AGH (Additional Files 3 and 7), and the Affymetrix 6.0 platform detected the only CNV tested on that platform that is very likely to be pathogenic (Patient 4821). None of the *de novo *CNV calls made only on the Affymetrix 6.0 platform occurred in regions that are known to be pathogenic for MR.

Two other validated *de novo *CNVs may be pathogenic. The first is a 152 Kb deletion of chromosome 21 that involves intersectin 1, which regulates endocytosis and dendritic spine development[37]. This deletion occurred in Patient 9609, who also has a pathogenic 728 Kb deletion of chromosome 22. The second possibly pathogenic CNV is a 378 Kb duplication that involves the distal portion of the 22q11.2 DGS/VCFS region (Patient 8327). A similar duplication was previously reported in a child and father whose cognitive ability was not clearly described[25].

## Discussion

In this study we compared the performance of various AGH systems for the clinical detection of pathogenic CNVs in children with MR. Previous studies that included more limited comparisons of AGH technologies found substantial differences in the CNV detection frequency between platforms, but some of these studies compared much lower resolution techniques and focused on larger CNVs[18] or compared lower- to higher-resolution arrays in an analysis that treated the higher-resolution findings as correct when discrepancy occurred[20]. In addition, most previous comparative studies were retrospective, focusing on the ability of various platforms to identify previously characterized CNVs. These studies only report detection of pathogenic CNVs and do not discuss findings with respect to the more frequent apparently benign variants[18-20].

Here we compared *de novo *CNVs identified on 3 platforms by analysing each child directly in relationship to his/her parent. This approach was the most cost-efficient to distinguish *de novo *and inherited CNVs using the comparative AGH methodology. However, using the parents as reference means that half of the hybridizations involve comparisons between samples from different sexes, and copy number estimates involving the X-chromosome(s) requires manual CNV identification by analysis of raw log_2 _ratios with the NimbleGen and Affymetrix 500 K software when there is a sex mismatch.

Each of the three main AGH platforms detected hundreds of autosomal CNVs in these 30 trios - an average of 34 CNVs per trio on NimbleGen arrays, of 22 CNVs on Aglient arrays, and 7 CNVs on Affymetrix 500 K arrays. What is most striking, however, is that 82% of the CNV calls were only made on one platform, suggesting a majority of false positive calls. As expected, most of the autosomal CNVs were inherited from one of the parents. However, many of the CNVs called in the child are probably not actually present in the child but rather represent a copy number change in the opposite direction in the parent, e.g., a copy number loss called in the child against the mother but not the father could actually be a copy number gain in the mother that was not transmitted to the child.

Overall, 146 autosomal *de novo *CNVs and 117 X-chromosomal CNVs were called on the 3 main platforms. 48 (32 *de novo *autosome and 16 X chromosome) of these CNVs were found on more than one platform. 10 *de novo *CNVs (9 autosome and 1 X chromosome) were called by all three main platforms. Genotype-phenotype correlations identified 7 CNVs that are likely to be pathogenic and 2 other CNVs that are good candidates to cause MR. All 9 of the pathogenic or possibly pathogenic CNVs were identified by each of the three main platforms.

Although we did not fully assess the Illumina Beadchips, their sensitivity appears to be similar to that of the Affymetrix 500 K arrays. Only 15 trios were assessed on the Affymetrix 6.0 platform. However, it is clear this platform produces many more CNV calls, although its detection rate for pathogenic and possibly pathogenic CNVs appears to be similar to that of the other SNP arrays. None of the additional *de novo *CNVs called on the Affymetrix 6.0 platform appears to be pathogenic.

The SMRT array only identified 5 of the 9 CNVs that were thought to be pathogenic or possibly pathogenic. One of the CNVs not identified was only 78 Kb in size and is probably below the resolution of the SMRT array. It is not clear why the other CNVs were not called by the SMRT array.

A number of differences exist among the platforms studied that may have contributed to the different results. Given the large number of genomic segments that were tested, there is a high probability that some of the *de novo *CNV calls are false positives in the proband and others are false negatives in a transmitting parent (i.e., the CNV is actually inherited, rather than *de novo*, in the proband). Differences in pre-processing, labelling, and hybridization protocols, which were performed according to the various manufacturers' specifications (see Additional File 1), could contribute to the occurrence of false negative and false positive calls. The lowest observed correlation between platforms was for smaller CNVs (data not shown) which highlights the importance of using probe number as a variable for identifying CNVs. Nevertheless, the lack of concordance in every single two-way comparison and the fact that there were *de novo *CNVs that were identified by one of the platforms that were not identified by any of the others make it very likely that neither optimization of the hybridization conditions nor optimization of the bioinformatic analysis parameters would produce perfect concordance.

Distinguishing pathogenic and benign CNVs is a major part of clinical CNV analysis and goes well beyond the software analysis performed on the data. We performed genotype-phenotype correlations for each *de novo *CNV using methods similar to those employed clinically, which have recently been discussed at length[4,12,13,22,23]. The size cut-off used in our study to assess pathogenicity (50 Kb) was arbitrary; however, almost all pathogenic CNVs detected by oligonucleotide AGH in recently reported studies of children with MR are much larger than 50 Kb[38,39].

Although the oligonucleotide or SNP-based AGH technologies studied detected all of the pathogenic or possibly pathogenic CNVs in the 30 MR trios studied, the need to manually assess CNVs on the sex chromosomes when there is a sex-mismatch with the NimbleGen and Affymetrix 500 K software is an important consideration when testing for MR or other genomic disorders in clinical service laboratories. Our results show that the tiling BAC array is less sensitive than the oligonucleotide or SNP-based arrays studied. In any case, the large number of apparently false positive CNV calls obtained with each of the platforms studied supports the need for validating all such calls with a different methodology before consideration of their possible pathogenicity in a clinical setting.

## Conclusions

It seems unlikely that any of the AGH platforms tested is completely right (or completely wrong) in its CNV calls. Clinical use of any AGH platform to detect pathogenic CNVs in children with birth defects continues to require considerable skill and experience.

## Competing interests

The authors declare that they have no competing interests. All authors read and approved the final manuscript.

## Authors' contributions

TT, AM, and SC and carried out the microarray studies. TT, AM, SC, AD, HQ, DV and JLM analysed the microarray data. TT and AM compared microarray platform data and TT performed the statistical analysis. TT, AM and JMF drafted the manuscript. DC, EM and TT performed molecular analysis to validated CNVs. BC and WLL provided array analysis software. GAR and JLM provided patients. PE, SL, JLM, JFM conceived the study and participated in study design.

## Pre-publication history

The pre-publication history for this paper can be accessed here:

http://www.biomedcentral.com/1755-8794/4/25/prepub

## Supplementary Material

Additional file 1 **Technical notes**. Detailed protocols for AGH and CNV size comparison between AGH platforms. In addition, there are detailed protocols for CNV validation and brief discussion of the difference between each AGH platform. Click here for file

Additional file 2 **Additional Table 5**. Summary of inherited CNVs identified in 30 MR trios identified by the Agilent 244 K, NimbleGen 385 K and Affymetrix 500 K arrays and correlation with Illumina Hap550, 32 K SMRT BAC and Affymetrix 6.0 platforms. Click here for file

Additional file 3 **Additional Table 6**. Summary of *de novo *CNVs identified in 30 MR trios identified by the Agilent 244 K, NimbleGen 385 K and Affymetrix 500 K arrays and correlation with Illumina Hap550, 32 K SMRT BAC and Affymetrix 6.0 platforms. Click here for file

Additional file 4 **Additional Table 7**. Summary of all CNV calls made by the Affymetrix 6.0 array in 15 MR trios studied. Click here for file

Additional file 5 **Additional Table 8**. Summary of all CNV calls made by the Illumina Hap500 beadchip in 30 MR patients studied. Click here for file

Additional file 6 **Additional Table 9**. Summary of all CNV calls made by the 32 K SMRT BAC tiling path array in 30 MR patients studies. Click here for file

Additional file 7 **Additional Table 10**. Summary of all X chromosome CNV in 30 MR trios identified by the Agilent 244 K, NimbleGen 385 K and Affymetrix 500 K arrays and correlation with Illumina Hap550, 32 K SMRT BAC and Affymetrix 6.0 platforms. Click here for file
