# Supplementary material for: Comparison of genome-wide array genomic hybridization platforms for the detection of copy number variants in idiopathic mental retardation
Source: BMC Med Genomics. 2011 Mar 25;4:25. doi: 10.1186/1755-8794-4-25 (PMC3076225; doi:10.1186/1755-8794-4-25)
Supplement: Additional file 1 — Technical notes. Detailed protocols for AGH and CNV size comparison between AGH platforms. In addition, there are detailed protocols for CNV validation and brief discussion of the difference between each AGH platform. [file 1755-8794-4-25-S1.DOC]

**AGH PROTOCOLS**

**Affymetrix 500K GeneChip**

The Affymetrix 500K GeneChip system (Affymetrix, Inc., Santa Clara, CA, USA) consists of two arrays (Nsp I and Sty I) containing probes for assaying a total of 500,568 SNPs, with a median spacing of 2.5 Kb across the genome. DNA samples were processed according to the instructions provided in the Affymetrix GeneChip® Mapping 500K Assay Manual. Briefly, 250 ng of high-quality genomic DNA were digested with Nsp I or Sty I and then ligated to Nsp I or Sty I adaptors. Adaptor-ligated restriction fragments were amplified by PCR and purified using a Clontech DNA Amplification Clean-Up Kit. A total of 90 µg of purified PCR product was fragmented and labeled with biotin before being hybridized to GeneChip® Human Mapping 250K Nsp or 250K Sty arrays in an Affymetrix Hybridization Oven 640. Arrays were washed with an Affymetrix Fluidics Station 450, and images were obtained using an Affymetrix GeneChip® Scanner 3000 7G and GeneChip® Operating Software version 1.4.

CNVs were detected using the Copy Number Analysis Tool (CNAT) (release cn-1.5.6_v3.2), which incorporates a Bayesian Robust Linear Model with the Mahalanobis distance classifier (BRLMM) algorithm to perform quantile normalization, followed by regression to reduce the PCR bias due to GC content and fragment length.

For trio analyses, we performed separate hybridizations for the mother, father, and child and then performed pair-wise comparisons of the normalized hybridization intensities for the child to the mother and of the child to the father *in silico.*

An HMM-based algorithm implemented in CNAT4 was used to estimate copy number for each SNP in the context of its surrounding SNPs. The Gaussian bandwidth was set to 0 Kb and a minimum of 5 probes was required to detect a CNV. The findings were visualized using GeneChip Genotyping Analysis Software (GTYPE) from Affymetrix.

**Agilent 244K Oligonucleotide Array**

The Agilent 244K oligonucleotide array (Agilent Technologies, Inc., Santa Clara, CA USA) contains 236,000 oligonucleotide probes plus 1,000 triplicates and 5,000 controls, with the median distance between probes in the genomic sequence being 8.9 Kb. All DNA samples were processed according to the instructions provided in the Agilent 244K Oligonucleotide Assay Manual. Briefly, 3 μg of high-quality genomic DNA were digested with *RsaI* and *AluI* and labeled with Cyanine-3 or Cyanine-5 using a random priming method. Two arrays were used for each trio, one in which the child’s DNA was hybridized against the father’s DNA, and another in which the child’s DNA was hybridized against the mother’s DNA. Equal amounts of the child’s and one parent’s DNA were labeled with opposite fluorochromes and hybridized to the array in an Agilent DNA Microarray Hybridization Oven. After the hybridization was completed, the arrays were scanned at 5µm resolution on an Agilent DNA Microarray Scanner.

Captured images were analysed with Feature Extraction v 9.1 and CGH Analytics 3.5.14 using the ADM-1 algorithm to identify regions with consistently high or low log_2_ ratio. This algorithm does not rely on a window size but rather samples immediately adjacent probes and uses the average normalized log_2_ ratios of all probes in a genomic interval to determine the deviation of the log_2_ ratio from its expected value of zero. A user-defined stringency threshold of 6, the recommended default value, was set.

Two other methods were also used to assist in the aberration calls. A centralization process was performed that assumes the log_2_ ratio values are centered at zero and re-centers the data by finding a constant value to add to or subtract from all log_2_ values. The program uses a default of a 10 probe window to average the log_2_ ratios. Also, a “fuzzy zero” method, which applies a global error mode to all aberrant intervals identified by the ADM-1 algorithm, was used to avoid erroneous aberration calls when the errors were correlated.

**NimbleGen 385K Oligonucleotide Array**

The 385K array produced by NimbleGen Systems, Inc., (Madison, WI, USA) contains 386,165 oligonucleotide probes with a median spacing of 6.2 Kb throughout the genome. DNA was sent to NimbleGen Systems, Inc. (Reykjavik, Iceland), where the hybridizations were performed. Briefly, 2 μg of high-quality genomic DNA were sonicated to produce a 500 bp to 2,000 bp smear on a 1% agarose gel. The DNA was labeled with Cyanine-3 or Cyanine-5 using a random priming method. Two arrays were used for each trio, one in which the child’s DNA was hybridized against the father’s DNA, and another in which the child’s DNA was hybridized against the mother’s DNA. 2μg each of the child’s and one parent’s DNA were labeled with opposite fluorochromes and hybridized to the array. After the hybridization was completed, the arrays were scanned at 5µm resolution on a GenePix 4000B Scanner.

Data from the hybridizations were analyzed using NimbleScan version 2.4.1 segMNT algorithm [60] with the following recommended parameters: 1) the minimum segment difference, which represents the minimum difference in the log_2_ ratio that two segments must exhibit before they are identified as separate segments, was set at 0.0; 2) the minimum segment length, which represents the minimum number of consecutive probes that must exhibit a change in log_2_ ratio in order to call a segment, was set at 2; and 3) the acceptance percentile, which represents the stringency with which initial segment boundaries are selected, was set at 0.999. Only segments with a mean absolute log_2_ ratio of 0.2 or more were considered for further analysis.

**Affymetrix 6.0 array**

The Affymetrix® Genome-Wide Human SNP Array 6.0 includes probes for the detection of over 906,600 SNPs and an additional 946,000 non-polymorphic oligonucleotides for the detection of copy number variation. The average inter-marker distance is less than 700 bp, so the average probe density is four times greater than that of the Affymetrix 500K array set.

The samples were prepared in the same manner as that used for the Affymetrix 500K array. CNVs were identified using the Genotyping Console 3.0.1 program. Since this program cannot perform pair-wise comparisons, we compared the results of each sample to a reference dataset generated by the combined results for the 30 parents in the 15 trios studied on the platform.

**Illumina Hap550 Beadchip**

The Illumina Human Hap550 Beadchip contains probes for 550,352 SNPs. Each Beadchip is composed of over 13 million 3-μm-diameter beads (with ~5-μm center-to-center spacing) with an average 20-fold redundancy for individual SNPs and a median probe spacing of 2.8 Kb. DNA from the proband in each trio was used for hybridization on the Illumina Human Hap550 Beadchips according to the manufacturer’s instructions. Briefly, approximately 500 ng of genomic DNA was used in a Whole-Genome Amplification (WGA) step. The amplified genomic DNA was fragmented to an average size of 300 bp using an endpoint enzymatic fragmentation protocol. After precipitation and resuspension in a hybridization buffer, the DNA was hybridized overnight to a Beadchip placed in a humidified chamber. On the next day, the Beadchips underwent single-base extension using labelled terminator nucleotides, which were then immunohistochemically stained to increase signal intensity. Finally, the Beadchips were imaged using a two-color confocal laser system with 0.8-μm resolution to identify both SNP alleles.

The intensities for each allele were extracted and normalized, and values for genotypes, allelic ratio (B-allele frequency) and logR ratio were calculated using an Illumina-supplied cluster file, which is based on a set of about 100 HapMap reference samples. The normalization algorithm adjusts for nominal offset, cross-talk, and intensity variations observed in the two-color channels.[61] CNV detection was done using PennCNV [20] with a threshold of 5 SNP-minimum per bin.

**SMRT 32K BAC Tiling Path Array**

Array CGH using Sub-Megabase Resolution Tiling-set (SMRT) human genomic BAC arrays (32,433 clones spotted in triplicate) was performed as previously described.[62] The analysis compared the patient to only one of the parents – the one of the same sex. 400 ng of DNA from the patient and 400 ng of DNA from the sex-matched parent were differentially labeled with Cyanine-3 or Cyanine-5 dCTPs (PerkinElmer, Woodbridge, Ontario, Canada) using a random priming protocol,[63] followed by array hybridization. The arrays were scanned at a resolution of 9.75 µm with an Arrayworx CCD scanner and analyzed using the Softworx array analysis platform (Applied Precision, Issaquah, WA). Visualization of the array data was performed using SeeGH software. [64]

Copy number changes were identified by a Hidden Markov Model using CNA-HMMer.[65] The user-defined probability factor for identifying a deletion or amplification was set at a threshold of 0.75 (range is 0-1): a CNV was called when the probability of deletion or amplification was greater than or equal to the threshold.

**Table 1:** Comparison of platform and settings used in identifying CNVs

| **Platform** | **Number of Probes** | **Median Probe Spacing** | **Number of Patients Tested** | **Number of Hybridizations Per Trio** | **Reference Used** | **Software Used** | **Algorithm Used** | **Min Probe** |
| --- | --- | --- | --- | --- | --- | --- | --- | --- |
| Affymetrix 500K | 500,568 | 2.5 Kb | 30 | 3 | Both Parents | Copy Number Analysis Tool V. 3.2 | HMM | 5 |
| Agilent 244K | 236,000 | 8.9 Kb | 30 | 2 | Both Parents | Feature Extraction V. 9.1 | ADM-1 | 3 |
| NimbleGen 385K | 386,165 | 6.2 Kb | 30 | 2 | Both Parents | NimbleScan V. 2.4.1 | segMNT | 2 |
| Affymetrix 6.0 | 1,852,600 | 0.7 Kb | 15 | 3 | Both Parents | Genotyping Console V. 3.0.1 | HMM | 1 |
| Illumina Hap550 | 550,352 | 2.8 Kb | 30 | 2 | Illumina Reference Set | PennCNV | Viterbi | 5 |
| SMRT 32K BAC | 32,433 | NA | 30 | 1 | Same-sex Parent | CNA-HMMer | HMM | 2 |

**DEFINING DE NOVO CNVS**

The analyses measured the strength of the hybridization signal obtained with the child’s DNA in comparison to that of each of his or her parent’s DNA. Each CNV call in which the child’s signal was less than that of the parent (called a “deletion” in the tally) could actually represent either a loss of copy number in the child or a gain of copy number in the parent. Similarly, each CNV call in which the child’s signal was greater than that of the parent (called an “amplification” in the tally) could actually represent either a gain of copy number in the child or a loss of copy number in the parent.

**Table 2. Summary of autosomal CNVs identified on the 3 main AGH platforms studied.**

|  | **Duplication** | | | | | | **Deletion** | | | | | |
| --- | --- | --- | --- | --- | --- | --- | --- | --- | --- | --- | --- | --- |
|  | **Number** | **Average Size*** | **Median Size*** | **Minimum Size*** | **Maximum Size*** | **IQR** | **Number** | **Average Size*** | **Median Size*** | **Minimum Size*** | **Maximum Size*** | **IQR** |
| Affymetrix 500K Only | 141 | 24 Kb | 16 Kb | 0.01 Kb | 681 Kb | 27 Kb | 50 | 51 Kb | 42 Kb | 0.03 Kb | 272 Kb | 58 Kb |
| Agilent 244K Only | 355 | 181 Kb | 25 Kb | 0.21 Kb | 569 Kb | 72Kb | 428 | 184 Kb | 25 Kb | 0.24 Kb | 13 Mb | 53 Kb |
| NimbleGen 385K only | 798 | 163 Kb | 82 Kb | 2.8 Kb | 843 Kb | 119 Kb | 743 | 184 Kb | 73 Kb | 2.8 Kb | 19 Mb | 116 Kb |
| Affymetrix 500K & Agilent 244K | 24 | 146 Kb | 97 Kb | 17 Kb | 1.2 Mb | 136 Kb | 15 | 92 Kb | 44 Kb | 13 Kb | 258 Kb | 147 Kb |
| Affymetrix 500K & NimbleGen 385K | 17 | 200 Kb | 113 Kb | 21 Kb | 664 Kb | 262 Kb | 21 | 83 Kb | 56 Kb | 17 Kb | 235 Kb | 99 Kb |
| Agilent 244K & NimbleGen 385K | 158 | 149 Kb | 51 Kb | 0.4 Kb | 1.7 Mb | 119 Kb | 171 | 156 Kb | 56 Kb | 1.8 Kb | 3.0 Mb | 110 Kb |
| All 3 | 76 | 268 Kb | 224 Kb | 12 Kb | 1.6 Mb | 177 Kb | 64 | 352 Kb | 117 Kb | 9.3 Kb | 9.8 Mb | 138 Kb |

IQR = Interquartile range

*The size of a CNV found on multiple platforms was determined as the common region of overlap. (Build 36).

**Table 3.** Summary of autosomal *de novo* CNVs identified on the three main AGH platforms.

|  | **Duplication** | | | | | | **Deletion** | | | | | |
| --- | --- | --- | --- | --- | --- | --- | --- | --- | --- | --- | --- | --- |
|  | **Number** | **Average Size*** | **Median Size*** | **Minimum Size*** | **Maximum Size*** | **IQR** | **Number** | **Average Size*** | **Median Size*** | **Minimum Size*** | **Maximum Size*** | **IQR** |
| Affymetrix 500K Only | 7 | 298 Kb | 101 Kb | 0.43 Kb | 1.6 Mb | 160 Kb | 12 | 78 Kb | 3.1 Kb | 0.03 Kb | 333 Kb | 144 Kb |
| Agilent 244K Only | 18 | 81 Kb | 22 Kb | 0.31 Kb | 1.6 Mb | 225 Kb | 24 | 84 Kb | 23 Kb | 0.40 Kb | 759 Kb | 57 Kb |
| NimbleGen 385K only | 14 | 331 Kb | 108 Kb | 43 Kb | 2.3 Mb | 286 Kb | 37 | 435 Kb | 180 Kb | 18 Kb | 2.5 Mb | 345 Kb |
| Affymetrix 500K & Agilent 244K | 0 |  |  |  |  |  | 3 | 175 Kb | 185 Kb | 132 Kb | 208 Kb |  |
| Affymetrix 500K & NimbleGen 385K | 0 |  |  |  |  |  | 0 |  |  |  |  |  |
| Agilent 244K & NimbleGen 385K | 9 | 474 Kb | 92 Kb | 47 Kb | 1.5 Mb | 1.3 Mb | 11 | 480 Kb | 132 Kb | 15 Kb | 3.0 Mb | 294 Kb |
| All 3 | 1 | 378 Kb |  |  |  |  | 8 | 2.0 Mb | 657 Kb | 78 Kb | 9.8 Mb | 1.2 Mb |

IQR = Interquartile range

*The size of a CNV found on multiple platforms was determined as the common region of overlap. (Build 36).

**CNV VALIDATION PROTOCOLS**

**FISH or MLPA Confirmation of Selected Autosomal CNVs**

Peripheral blood lymphocytes were prepared to obtain chromosome preparations according to standard protocols.[66] Probes for FISH confirmation were selected on the UCSC genome browser within the RP11 library on genome build 36. FISH was performed on metaphase and, for duplications, interphase chromosomes according to standard protocols.[66]

Multiplex Ligation-dependant Probe Amplification (MLPA) was conducted according to the manufacturer's recommendations. Briefly, the patient's DNA was diluted in PCR-grade water, and the quality was assessed by Nanodrop. The hybridization solution (SALSA probe-mix and MLPA buffer) was added to a final DNA concentration of 60ng/μl. DNA was denatured at 90C and hybridized for 16-20 hours at 60C. Ligation was performed at 54C for 15 minutes, and the ligated products were subsequently denatured at 98C for 5 minutes. The ligated products were then amplified by PCR (SALSA PCR buffer, PCR-primers and polymerase). The PCR products were run on an ABI-3130 sequencer for fragment analysis. Normalization of the data and analysis of the MLPA results were conducted using Coffalyser v3.5 software provided by MRC Holland.

**Confirmation of Selected X Chromosomal CNVs**

PCR was performed to confirm deletion CNVs in males. Primers were designed within the MR candidate gene within the identified CNV while avoiding benign polymorphisms listed in the DGV (Table S8). The patient's DNA was diluted in PCR-grade water and the quality was assessed by Nanodrop. PCR was performed using a final DNA concentration of 100 ng/μl, and the PCR product was visualized on a 2% agarose gel stained with ethidium bromide. The absence of a band in the child and the presence of a band in the mother, father and unrelated controls with the test primers and the presence of a band in all individuals for primers designed for the dystrophin (DMD) gene was considered a confirmed deletion.

Amplifications CNVs were validated by qPCR using SYBR Green (Applied Biosystems). Primers were designed using Primer Express (Applied Biosystems) (Table S1), and qPCR was performed on ABI7000 using both parents and an unrelated male and female as copy number controls and glucose-6-phosphate dehydrogenase (G6PDH) gene as a locus control.

**Table 4:** qPCR and PCR Primers used for validation

| Gene | CNV | Forward Primer | Revers Primer | P-length | NCBI Assembly | Test Method |
| --- | --- | --- | --- | --- | --- | --- |
| ARSD | DEL | TCACGTCCAACCTGGATGTG | CAAAGACCCGCTTCAGTTCTACA | 77 | 36.3 | PCR |
| DMD5 | DEL | AAACATGGAACATCCTTGTGGGGAC | CATTCCTATTAGATCTGTCGCCCTAC | 547 | 36.3 | PCR |
| DUSP21 | AMP | GCCAACGACAAACTCCTTCTG | ACTTCCACCGAGGCATTGAC | 65 | 36.3 | qPCR |
| G6PDH | CNT | CCCTGCACAGTGTTACCCTT | CCTTAGCTGTCCTCACCGAC | 236 | 36.3 | qPCR |
| GPM6B | DEL | CCAATGTTCCTCACGAGCAA | AGCTGTGAAAGTAGGATTGGATAACC | 97 | 36.3 | PCR |
| GRIA3 | DEL | AGCTAATTGCCACATTGCTGAGCC | GGCTTTCAAATGACAGACAGGCCATC | 194 | 36.3 | PCR |
| HDAC8 | DEL | TGTAGACTGCCTGGAACGAAGA | GAGACTGATCTGTAGTGAAAGCATGAG | 98 | 36.3 | PCR |
| KAL1 | DEL | AAGATGGAGGCAGGAGAGCAGTTT | TCTCCTTGCCAATGTCCCTGTCTT | 326 | 36.3 | PCR |
| LDOC1 | DEL | TGAGGAGGCTGAT TAGGAATGC | GGCGTCTTACATGCTCGTGAA | 77 | 36.3 | qPCR |
| LHFPL1 | DEL | AGCAGTGGGCCTGACATTTAA | GCCGCCAGTGACAATTTATCTC | 149 | 36.3 | PCR |
| MAGEH1 | DEL | TCATCTTCATCATGGGCAACAG | TCCAGGCTGCATTCCTAACTTC | 79 | 36.3 | PCR |
| PCDH11X | DEL | AAACGTCCTGATAGGCGACTTGTTGA | TGTTGCTGGGAACAATGGTGCATTA | 319 | 36.3 | PCR |
| PHF8 | DEL | ATGAGTGGGACTGGTAGCCAAACA | TCCAACAGAACGTTGGGAAGACGA | 431 | 36.3 | PCR |
| PLXNA3 | DEL | TCCCAGTGAAGGTTCTCAACTGTGAC | TCACACTCGATCTTGGTGGTGACATC | 350 | 36.3 | PCR |
| SMC1A | DEL | TCCACAACTGAGAGAACCTGGCTT | TGAGCTATGGGCATTTCAGTCCCT | 378 | 36.3 | PCR |
| UBQLN2 | AMP | AATCATCAAAGTCACGGTGAAGAC | CTTAAACTGCTGAACCGAGCTGTT | 82 | 36.3 | qPCR |
| ZNF449, ZNF75 | DEL | AATTCTCTGGGTCCTCACAACCTGTC | TTTACTCCTTCTTGGCCTACCTGCTG | 453 | 36.3 | PCR |
| ZNF630 | AMP | CCTGAGCAAAGGCGTGGCGA | CGGGTCCTGACGCCAAGCAG | 230 | 36.3 | qPCR |

**DIFFERENCES BETWEEN PLATFORMS**

Sample processing differs between the NimbleGen and Agilent arrays in comparison to the Affymetrix arrays. The Agilent and NimbleGen procedures use random priming to label the samples without PCR amplification, while the Affymetrix protocol requires a large number of PCR cycles to amplify and label the DNA. In any case, problems with labeling efficiency due to random priming or preferential amplification of one allele could generate false positive or false negative calls.

In addition, the design of the probes put on the arrays is different. Both the Agilent and NimbleGen arrays use longer (50-75 basepairs) non-polymorphic oligonucleotide probes, while the Affymetrix platform contains shorter (25 basepairs) probes of varying sequence designed to identify SNPs. The longer probes may allow for hybridization despite a small amount of sequence mismatch. Alternatively, unknown SNPs present under the DNA priming sites may interfere with primer binding and prevent hybridization and therefore not identify a potential CNV in that region.

The Affymetrix 500K platform identified significantly fewer CNVs than the Agilent or NimbleGen platforms. The majority of CNVs identified on at least 2 platforms were identified by Agilent and NimbleGen, which is not surprising given the larger number of CNVs identified on these 2 platforms and the greater similarity of these non-polymorphic oligonucleotide arrays to each other and their differences from the Affymetrix 500K SNP array. The lower number of CNV calls on the Affymetrix 500K platform is probably due in part to poor probe coverage of gene-poor regions, telomeres, centromeres and genomic regions that are rich in segmental duplications. Over 40% of the *de novo* CNVs called by the Agilent or NimbleGen platforms could not have been called on the Affymetrix 500K platform because fewer than 5 probes (our lower limit for making a call) were present in the involved region. Another 6% of the CNVs that were called as *de novo* on the Agilent or NimbleGen platforms were found to be inherited from one parent on Affymetrix 500K AGH and probably represent false negative calls in the parent on the Agilent or NimbleGen platforms.

Another critical difference among the technologies studied is the reference DNA used for comparison (See Supplementary methods). Very important differences also exist among the platforms in the software algorithms used for feature recognition, normalization, smoothing and CNV calling. The default settings also vary and could affect the CNV calls. It is likely that better performance could be achieved with each of the platforms by optimizing the hybridization conditions and analyses.
